# Highly bioavailable curcumin preparation with a co‐grinding and solvent‐free process

**DOI:** 10.1002/fsn3.1930

**Published:** 2020-10-07

**Authors:** Yiying Lu, Mengting Lin, Jiancheng Zong, Lei Zong, Zhen Zhao, Shanglong Wang, Zengliang Zhang, Min Han

**Affiliations:** ^1^ Institute of Pharmaceutics College of Pharmaceutical Sciences Zhejiang University Hangzhou People’s Republic of China; ^2^ Chenland Nutritionals, Inc. Irvine CA USA; ^3^ Pathology and Laboratory Medicine Weill Cornell Medicine New York NY USA; ^4^ Traditional Chinese Medicine College Inner Mongolia Medical University Hohhot China

**Keywords:** aqueous solubility, co‐grinding, curcumin, oral bioavailability, Poloxamers

## Abstract

Curcumin (Cur.) is a natural product isolated from the rhizome of *Curcuma longa,* with a variety of biological and pharmacological activities in food and pharmaceutical products. However, curcumin's poor solubility in water greatly limits its bioavailability and clinical applications. In this study, co‐grinding curcumin with food additives produced a mixture, which was evaluated for the solubility in water, dissolution, material morphology, in vivo bioavailability, cell uptake and entry mechanism. We tested 9 food additives in total and found that poloxamers performed the best. The 2 co‐grinding mixtures Cur./Kolliphor^®^ P407 and Cur./Kolliphor^®^ P188 with high drug loading at 65.5% significantly improved the curcumin aqueous solubility, subsequently increased its intestinal epithelial cell uptake and oral bioavailability. The relative bioavailabilities for the 2 co‐grinding mixtures were 309% and 163%, respectively, compared with curcumin API. Co‐grinding process has a broad application prospect and is suitable for industrial production.

## Graphical abstract

The 2 co‐grinding mixtures Cur./Kolliphor® P407 and Cur./ Kolliphor® P188 with high drug loading at 65.5% significantly improved the curcumin aqueous solubility and oral bioavailability. The relative bioavailabilities for the 2 co‐grinding mixtures were 309% and 163%, respectively, compared to curcumin API.

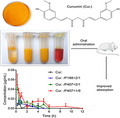

## 1 INTRODUCTION

As a natural hydrophobic polyphenol extracted from the rhizome of the herb *Curcuma longa*, curcumin (diferuloylmethane; 1,7‐bis[4‐hydroxy‐3‐methoxyphenyl]‐1,6‐heptadiene‐3,5‐dione) has a wide range of biological and pharmacological activities including anti‐inflammatory (Menon & Sudheer, 2007), anti‐oxidation (Bojorges et al., 2020; Wang et al., 2008), and antitumor (Jaggi et al., 2007; Mukherjee et al., 2016) properties. Some anticancer drugs can also be used in conjunction with curcumin to reduce their toxicity (Jafarinezhad et al., 2019). Studies have revealed therapeutic effects of curcumin in many related diseases such as rheumatoid arthritis (Chandran & Goel, 2012), inflammatory bowel diseases (IBD)(Gong et al., 2018), diabetic complications (Parsamanesh et al., 2018), hypertriglyceridemia (Sahebkar, 2014), and ischemic injury (Sahebkar, 2010). Commercially available curcumin generally contains demethoxycurcumin and bisdemethoxycurcumin in addition to curcumin. As a widely used food‐coloring agent in India and Southeast Asia, curcumin is safe and tolerable for volunteers to take as high as 12 g per day (Lao et al., 2006). However, the poor solubility in water (Mirzaei et al., 2017), instability at physiological and alkaline pH (Kharat et al., 2017), and rapid metabolism (Metzler et al., 2013) result in the low oral bioavailability (OB) of curcumin and thus limit its applications.

Various strategies have been explored to increase the OB of curcumin with the help of curcumin–phospholipid complex (Maiti et al., 2007), polymeric micelle (Letchford et al., 2008; Ma et al., 2007), liposomes (You et al., 2014), microemulsion, nanoemulsion, self‐emulsifying drug delivery systems (SEDDS) (Bergonzi et al., 2014; Yan et al., 2011), nanoparticles (Cui et al., 2019; Ji et al., 2016), and OB enhancer‐like piperine (Shoba et al., 1998). Although these methods can improve the OB of curcumin to some extent, their wider application is hindered by some disadvantages exposed in commercial production, such as complicated prescription and sophisticated process, high cost price, and low drug‐loading capacities. For example, when completely dissolved in microemulsion/nanoemulsion/SEDDS formulations, the amount of curcumin is limited by its solubility in surfactant–oil mixtures. Being incorporated in soft gelatin capsules, the final cost of microemulsion/nanoemulsion/SEDDS preparations will raise. With further solidifying of these formulations, the concentration of curcumin in such preparations decreases even more, resulting in larger volume in these solid dosage forms (including tablets or capsules) to provide a therapeutic dose, thereby reducing patient compliance. Furthermore, some frequently used excipients have irritation to gastric mucosa (such as some absorption promoters and a large number of surfactants). Similarly, the preparation of curcumin polymeric micelle, liposomes, nanoparticles, and solid dispersions usually involves the utilization of organic solvents and drying process, such as spray‐drying or freeze‐drying with excipients, which leads to increase in production cost and commercial price (Madhavi & Kagan, 2014). Some of the steps are also environmentally unfriendly. Therefore, further research and improvement are required.

Poly (ethylene oxide)–poly (propylene oxide) amphiphilic block copolymers (Poloxamers) serve well as pharmaceutical excipients because of their highly tunable association properties, low toxicity, and ability to functionalize (Bodratti & Alexandridis, 2018). Poloxamer 407 and Poloxamer 188 are the most widely used excipients in the field of pharmaceutical preparations. They usually served as matrix, solvent, stabilizer, emulsifier, absorption promoter, solid dispersion carrier, *etc.,* to control drug release, improve drug stability, increase the solubility of insoluble drugs, and enhance drug bioavailability. So they are considered as ideal carriers for hydrophobic drugs including curcumin.

Here, we report a new and simple co‐grinding process via solvent‐free approach to improve the OB of curcumin with high drug loading at 65.5%. For the first time, poloxamer with good safety is employed as the excipient to mix and co‐grind with curcumin to obtain a solid curcumin raw material. Compared with curcumin in specific dosage forms, this preparation can be used for the further processing of various dosage forms such as tablets, capsules, granules, or pills with a broader application prospect. What's more, being simple and cost‐effective, the prescription process is more suitable for industrial production.

## 2 MATERIALS AND METHODS

### 2.1 Chemicals

Curcumin API (95%, CUR) was provided by JIAHERB Phytochem Co., Ltd. (China). Standard Curcumin was purchased from Dingrui Chemical Co., Ltd. (Shanghai, China). Chitosan (MW: 50k, Zhejiang Jinke Pharmaceutical Co., LTD.), Kolliphor^®^ P407(P407), Kolliphor^®^ HS15(HS15), Kolliphor^®^ ELP(ELP), Soluplus^®^, and Kolliphor^®^ P188(P188) were obtained from BASF (Shanghai, China). Fetal bovine serum was purchased from Gibco (America), 0.25% EDTA‐pancreatin was purchased from Senrui Biotechnology Co., Ltd. (Zhejiang, China). R1640 culture solution, DMEM high‐sugar culture solution, and penicillin–streptomycin mixed solution (100 ×) were purchased from Ginuo Biological Pharmaceutical Co., Ltd. (Zhejiang, China). Nitrendipine was purchased from Mclean Biochemical Technology Co., Ltd. (Shanghai, China). Nitrendipine and mobile phase are of chromatographic purity; all the other chemicals were of analytical reagent grade unless stated otherwise.

### 2.2 Cells and animals

Human colon cancer cells Caco‐2, HT‐29, and human Burkitt's lymphoma cell Raji‐B were purchased from Cell Bank of the Chinese Academy of Sciences (Shanghai, China). Male Sprague Dawley (*SD*) rats weighed 200g were purchased from Shanghai Laboratory Animal Center (SLAC) (Shanghai, China). All in vivo experiments were performed strictly according to guidelines that were evaluated and approved by the Ethics Committee of Zhejiang University.

### 2.3 Establishment of HPLC analytical method for curcumin in vitro

An appropriate amount of curcumin standard was weighed and dissolved in methanol to prepare curcumin methanol solution with a concentration of 50 mg/ml. Zero was corrected with methanol, UV scanning was performed within the wavelength range of 200–800 nm, and the maximum absorption wavelength was selected as the detection wavelength.

By exploring different mobile phases and ratios, curcumin was required to be separated from methoxycurcumin and dimethoxycurcumin at baseline, and the peak time was less than 25 min (Syed et al., 2015).

A proper amount of curcumin API was weighed and dissolved in methanol to prepare mother liquor with a concentration of 1 mg/ml, which was successively diluted with methanol to different concentrations ranging from 500 μg/ml to 0.122 μg/ml. The sample was injected to HPLC according to the chromatographic conditions explored above, and the peak time and peak area were recorded, respectively. The standard curve was drawn with the concentration as the horizontal coordinate and the peak area as the vertical coordinate.

### 2.4 Determination of solubility

The solubility of curcumin was studied in different solvents, including ultrapure water, 0.1 M hydrochloric acid, and phosphate buffer (PBS) with a pH of 6.8. Excessive curcumin API was added to 10 ml centrifuge tubes containing different solvents and were oscillated for 72 hr at 100 rpm in a constant temperature shock box at 37°C. The supernatant was removed and analyzed by HPLC after high‐speed centrifugation at 13,000 rpm for 10 min.

### 2.5 Determination of oil–water partition coefficient

The absorption, distribution, metabolism, and excretion (ADME) of the drug in the body are closely related to its water solubility and lipid solubility. Therefore, the oil–water partition coefficient (P) of curcumin was measured, that is, the ratio of the drug concentration in the oil phase and water phase. In this experiment, the octanol–water system was adopted. By measuring the drug concentration c_1_ in octanol saturated with water and c_2_ in water saturated with octanol (the two phases have the same volume), formula *p* = c_1_/c_2_ was used to calculate the oil–water partition coefficient.

Specifically, measure 10.00 ml water‐saturated octanol solution of curcumin API in a 50 ml centrifuge tube with a pipette accurately, and then measure 10.00 ml water saturated with octanol. After being sealed, the centrifugal tube was oscillated for 72 hr at 100 rpm in a constant temperature shock box at 37°C. Finally, the oil phase and water phase were separated, the oil phase was diluted with methanol and analyzed by HPLC while the water phase was analyzed directly by HPLC.

### 2.6 Solubilization effect of different excipients

The solubilization effect of 9 excipients (the mass ratio of curcumin API and excipients is 1:1) was investigated to screen excipients with superior solubilization effect for the next experiments. Different excipients include PVP, Chitosan, Kolliphor^®^ P407, PEG 6,000, Phospholipid, Kolliphor^®^ HS15, Kolliphor^®^ ELP, Soluplus^®^, and Kolliphor^®^ P188 were weighted into separate 2 ml test tubes, and equal mass of curcumin API was added. Two small steel balls were added to each tube, which was placed in a grinder for grinding at a frequency of 60 Hz for 2 min (Automatic Grinding Instrument (JXFSTPRP24, Shanghai Jingxin Industrial Development Co., Ltd.)). The drug‐loading capacity (DL) is calculated according to the formula DL = m _Curcuminoids_/ (m _Curcuminoids_ + m _Excipient_) *100%, m, mass.

70 mg of each ground powder was placed in a 10‐ml centrifuge tube with 7 ml ultrapure water in it, respectively. The centrifuge tube was oscillated for 72 hr at 100 rpm in a constant temperature shock box at 37°C. The upper liquid was centrifuged at 13,000 rpm for 10 min, and the supernatant was taken for HPLC analysis.

### 2.7 Scanning electron microscope (*SEM*)

Scanning electron microscope can obtain more abundant characteristic information of sample surfaces by scanning the sample with electron beams. *SEM* was used to observe the surface characteristics of the curcumin API and its mixture after grinding with excipients, so as to see whether there is a difference in physical morphology between them.

### 2.8 Dissolution test in vitro

Two kinds of excipients with superior solubilization effect under 2.6 were selected for the dissolution test. Superior solubilization means the solubility of curcumin is above 10 μg/ml. The dissolution medium was 900 ml of a 0.5% sodium dodecyl sulfate (SDS) solution. The rotation speed is 75 rpm, and the temperature is 37°C. 20 mg of Soluplus^®^ curcumin co‐grinding mixture, Kolliphor^®^ P188 curcumin co‐grinding mixture, Kolliphor^®^ P407 curcumin co‐grinding mixture, and 10 mg curcumin API were put into different dissolution tanks, which were sampled 3 ml in the same position with a sampling needle for 5 min, 10 min, 20 min, 40 min, 1 hr, 1.5 hr, 2 hr, 3 hr, 4 hr, 6 hr, 8 hr, and immediately supplemented dissolution cylinder with isothermal and isovolumetric media (0.5% SDS). The removed samples were filtered by 0.22μm microporous filter membrane and analyzed by HPLC.

### 2.9 Cell experiment

#### 2.9.1 Cell culture

Caco‐2 cells were cultured in DMEM high‐glucose culture solution (containing 10% fetal bovine serum, 1% penicillin–streptomycin mixed solution, 1% nonessential amino acid) and incubated in an incubator at 37°C and 5% CO_2_. The solution was changed every two days and digested with 0.25% EDTA–trypsin when the growth was over 80%. HT‐29 cells and Raji‐B cells were cultured in R1640 culture solution (containing 10% fetal bovine serum and 1% mixed solution of penicillin–streptomycin) and incubated in an incubator at 37°C and 5% CO_2_. The solution was changed every two days. When the growth was over 80%, they were passaged at 1:5.

#### 2.9.2 Cell tight junction test (ZO‐1 cell immunofluorescence test)

Caco‐2 cells at logarithmic growth stage were seeded in confocal dishes at a density of 2 × 10^5^ cells/ml. The culture solution was changed every other day during the first week and daily from the second week. After 14 days of culture under the condition of 37°C and 5% CO_2_, the culture solution was removed and the Caco‐2 cells were washed with PBS for three times, and 1 ml of the drug‐containing culture solution was added there to have a curcumin concentration of 20 μg/ml (drug in group 2: curcumin; drug in group 3: curcumin and chitosan). After 4 hr of co‐incubation, the drug‐containing solution was removed, Caco‐2 cells were washed with PBS for three times, and 4% paraformaldehyde was used to fix the cells for 20 min. After the fixation was completed, PBS was used to wash cells twice for 5 min each time. The immunostaining blocking solution was added and blocked for 60 min. After blocking, PBS was used to wash cells twice for 5 min each time. The ZO‐1 primary antibody was added and incubated at 37°C for one hour. Washed with PBS 3 times for 5 min each time. The goat anti‐rabbit secondary antibody was added and incubated at 37°C for one hour, and washed with PBS 3 times for 5 min each time. 1 ml of ready‐to‐use DAPI was added to each well, and after incubation for 8 min, PBS was used to wash cells 3 times. Cells were observed under a confocal microscope.

#### 2.9.3 Cell uptake (qualitative)

Caco‐2 cells at logarithmic growth stage were seeded in confocal dishes at a density of 4 × 10^4^ cells/ml. The culture solution was changed every other day during the first week and daily from the second week. After 14 days of culture under the condition of 37°C and 5% CO_2_, the culture solution was removed, and cells were cultured for 4 hr with a drug‐containing serum‐free solution (curcumin concentration: 20 μg/ml). After three times of washing with PBS in dark, the fluorescence of intracellular curcumin in different groups was observed and photographed under a confocal microscope. The stronger the fluorescence was, the more the uptake was indicated (Kunwar et al., 2008).

#### 2.9.4 Cell uptake (quantitative) and cell entry mechanism study

Confocal microscope can visually observe the relative amount of curcumin ingested by cells, but it cannot accurately show the difference of curcumin ingested by different groups of cells within 4 hr, so quantitative study on the intake of curcumin is needed. To calculate the cell uptake rate of drugs, flow cytometry with high sensitivity was used to determine the fluorescence value of curcumin for quantitative determination.

Here, we used a three‐cell model: Caco‐2 cells and HT‐29 cells at logarithmic growth stage were seeded in 6‐well plates at densities of 7 × 10^4^ cells/ml and 3 × 10^4^ cells/ml, respectively. The culture solution was changed every other day during the first week and daily from the second week. The culture lasted for 14 days. In the last 3 days, Raji‐B cells at logarithmic growth stage were inoculated at the density of 1 × 10^4^ cells/ml. After 14 days, the culture solution was removed and the drug‐containing serum‐free solution (curcumin concentration: 20 μg/ml) was added into plates and cultured cells for 4 hr. After that, the cells were removed in dark, washed with PBS for three times, and digested with 0.25% EDTA–trypsin. The serum‐containing culture solution was added to terminate the digestion. The cells were centrifuged at 1,200 rpm for 5 min, washed with PBS for two times, and then resuspended and sieved. Using the characteristics of curcumin autofluorescence, the cell uptake rate of curcumin was quantitatively determined by flow cytometry (488 nm excitation, blue light, FL1, 530 ± 15 nm, FITC/GFP).

To explore cell entry mechanism, after 14 days’ culture, the culture solution was removed and the inhibitor was added for half an hour (sodium azide concentration was 1 mg/ml, ketoconazole concentration was 10 μg/ml, and verapamil concentration was 300 μg/ml), and then, the inhibitor was removed, and curcumin‐containing serum‐free solution (curcumin concentration: 20 μg/ml) was added. The following steps including cell digesting, washing, and detection by flow cytometry are the same as above.

### 2.10 Animal experiment

#### 2.10.1 Establishment of HPLC analytical method for curcumin in blood samples

The nitrendipine was used as an internal standard to quantify curcumin in plasma. By exploring different mobile phases and ratios, curcumin was required to be separated from methoxycurcumin and dimethoxycurcumin at baseline, and the peak time was less than 25 min.

#### 2.10.2 Pharmacokinetics study

The male Sprague Dawley rats were divided into 4 groups (*n* = 3). Group 1 was administered orally 50 mg/kg body weight (BW) curcumin solution (preparation method: curcumin and P407 with a mass ratio of 1:9 were completely dissolved in ethanol. Rotary evaporation under reduced pressure was used to remove ethanol for later use, and saline was added to obtain a clear and transparent solution before gavage administration). Group 2 received 75 mg/kg BW grinding mixture of curcumin and P407 dissolved in saline, and group 3 received 75 mg/kg BW grinding mixture of curcumin and P188 dissolved in saline. Group 4 was administrated orally 50 mg/kg BW curcumin API dissolved in saline. In summary, all rats were given 50 mg/kg BW of curcumin in different preparations.

After administration, a 0.5 ml blood sample was collected into tubes containing heparin sodium from infraorbital venous plexus in 15 min, 30 min, 45 min, 1 hr, 2 hr, 3 hr, 4 hr, 5 hr, 6 hr, and 9 hr intervals. After centrifugation at 4,000 rpm for 5 min, 200 μL of plasma was taken and added to 100 μL nitrendipine dissolved in ethyl acetate solution (250 μg/ml), and then, 1 ml of ethyl acetate was added and mixed in a vortex for 2 min. The supernatant (organic layer contained curcumin and nitrendipine) was obtained by centrifugation at 12,000 rpm for 5 min. After evaporating the ethyl acetate, 100 μL of methanol was used to redissolve it. The standard curve solution was treated using the same method.

## 3 RESULTS AND DISCUSSION

### 3.1 Curcumin HPLC analytical method

**FIGURE 1:**
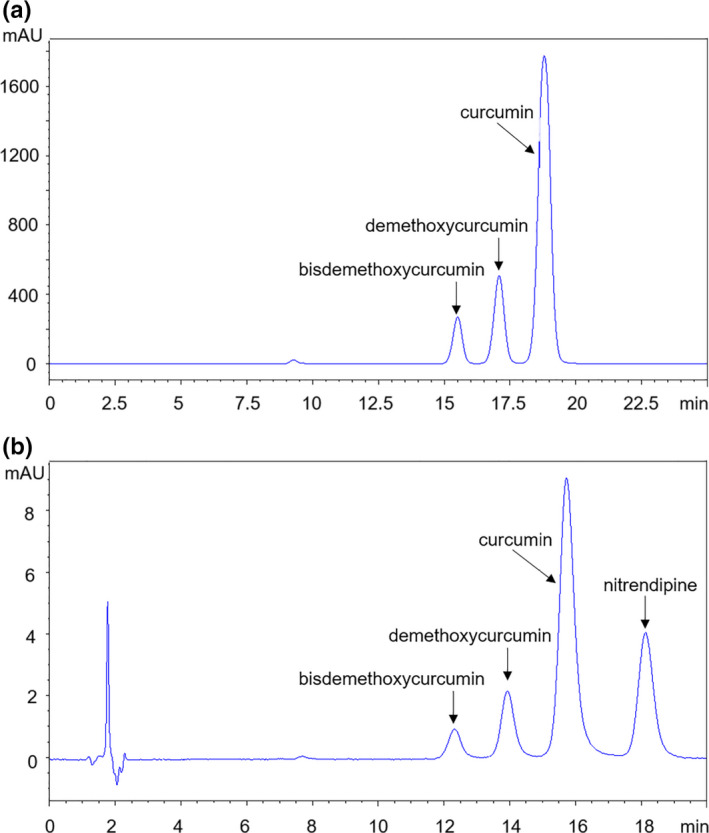
The liquid chromatography diagram of curcumin API. The purity of curcuminoids was 98.3%, and the purity of curcumin is 72.4%. (a) The analytical method for in vitro samples. Composition of mobile phase: acetonitrile: methanol: water (0.3% chromatographically pure acetic acid) (v:v:v) = 40:12:48. The peak time of curcumin, demethoxycurcumin, and bisdemethoxycurcumin was 18.803/17.081/15.507 min, respectively. (b) The analytical method for plasma samples. Curcumin and nitrendipine were extracted from plasma by ethyl acetate. After evaporating the ethyl acetate, methanol was used to redissolve it. Then, the solutions containing curcumin and nitrendipine were analyzed by HPLC. Composition of mobile phase: water (5% chromatographically pure acetic acid): acetonitrile = 55:45. The peak time of nitrendipine, curcumin, demethoxycurcumin, and bisdemethoxycurcumin was 18.133/15.726/13.931/12.319 min, respectively

The optimized condition of the curcumin HPLC analytical method is described here: chromatographic column: partisil 5μm ODS, 150*4.6nm; detection wavelength: 421nm; flow rate: 1.0 ml/min; column temperature: 25°C; injection quantity: 20μL; acetonitrile: methanol: water (0.3% chromatographically pure acetic acid) (v: v: v) =40:12:48 for vitro sample analysis and water (5% chromatographically pure acetic acid): acetonitrile = 55:45 for plasma sample analysis. The liquid chromatography diagram is shown in Figure 1.

### 3.2 Solubility and oil–water partition coefficient of curcumin

The solubility of curcumin in water, PBS, and 0.1mol/L hydrochloric acid was 1.622, 0.310, and 0.675 μg/ml, respectively. The water solubility of curcumin was proved to be extremely poor.

The solubility of curcumin was 0.103 μg/ml in octanol‐saturated water and 1,487.0 μg/ml in water‐saturated octanol. *p* = 14,504.67, so the calculated oil–water partition coefficient of curcumin log P was 4.16. The results showed that curcumin had strong lipophilic property and poor hydrophilicity. It suggests that improving the solubility of curcumin may be a key factor in improving the oral absorption and bioavailability of curcumin.

### 3.3 Solubilization effect of different excipients

When directly mixed together without grinding process, the complex of curcumin and excipients may lack stability due to nonuniformity in their density, particle size and surface properties, especially in transit or after long‐term storage. Now, the process of mixing and grinding curcumin with excipients, on the one hand, averages their differences in particle size and reduces the possibility of material layering. On the other hand, the two are brought into full contact by grinding, so that the surface static of curcumin can be decreased by the surface activity of excipients, which is also beneficial to maintaining the stability of the mixture. Besides, the close combination of excipients and curcumin can form a locally water‐soluble microenvironment around curcumin, improving the solubility of curcumin and facilitating dissolution during the digestion process. The dissolution experiment in vitro (Fig. S1) showed that the release of curcumin in co‐grinding mixture (micronized) is faster than which without grinding (directly mixing, nonmicronized), and the dissolution difference was particularly obvious in the first hour. The rapid dissolution of curcumin brought by the grinding process was beneficial to its absorption in vivo. A preliminary study showed that the homogeneous material with good dispersion can be obtained by controlling certain parameters as follows: 2 small steel balls, frequency of 60 Hz, and a time of 2 min.

The solubility changes of curcumin after co‐grinding with different excipients were shown in Table 1. The c in the table represents the concentration of curcumin. The solubility of curcumin in group P407, HS15, ELP, Soluplus, and P188 increased significantly compared with 1.622 μg/ml (the solubility of curcumin in water, according to section 3.2). However, as HS15 and ELP are viscous semisolids at room temperature, commonly used in liquid and semisolid preparations, physical stability will be poor if used in solid formulations, further exploration and usage were abandoned.

**TABLE 1:** Solubility of curcumin in different co‐grinding mixture

Excipients	Con./(μg/ml)	Excipients	Con./(μg/ml)	Excipients	Con./(μg/ml)
PVP	0.503	PEG 6,000	1.058	ELP	27.280
Chitosan	0.097	Phospholipid	0.513	Soluplus	200.028
P407	88.125	HS15	44.515	P188	24.522

Abbreviation: Con., the concentration of curcumin.

### 3.4 Scanning electron microscope (*SEM*)

**FIGURE 2:**
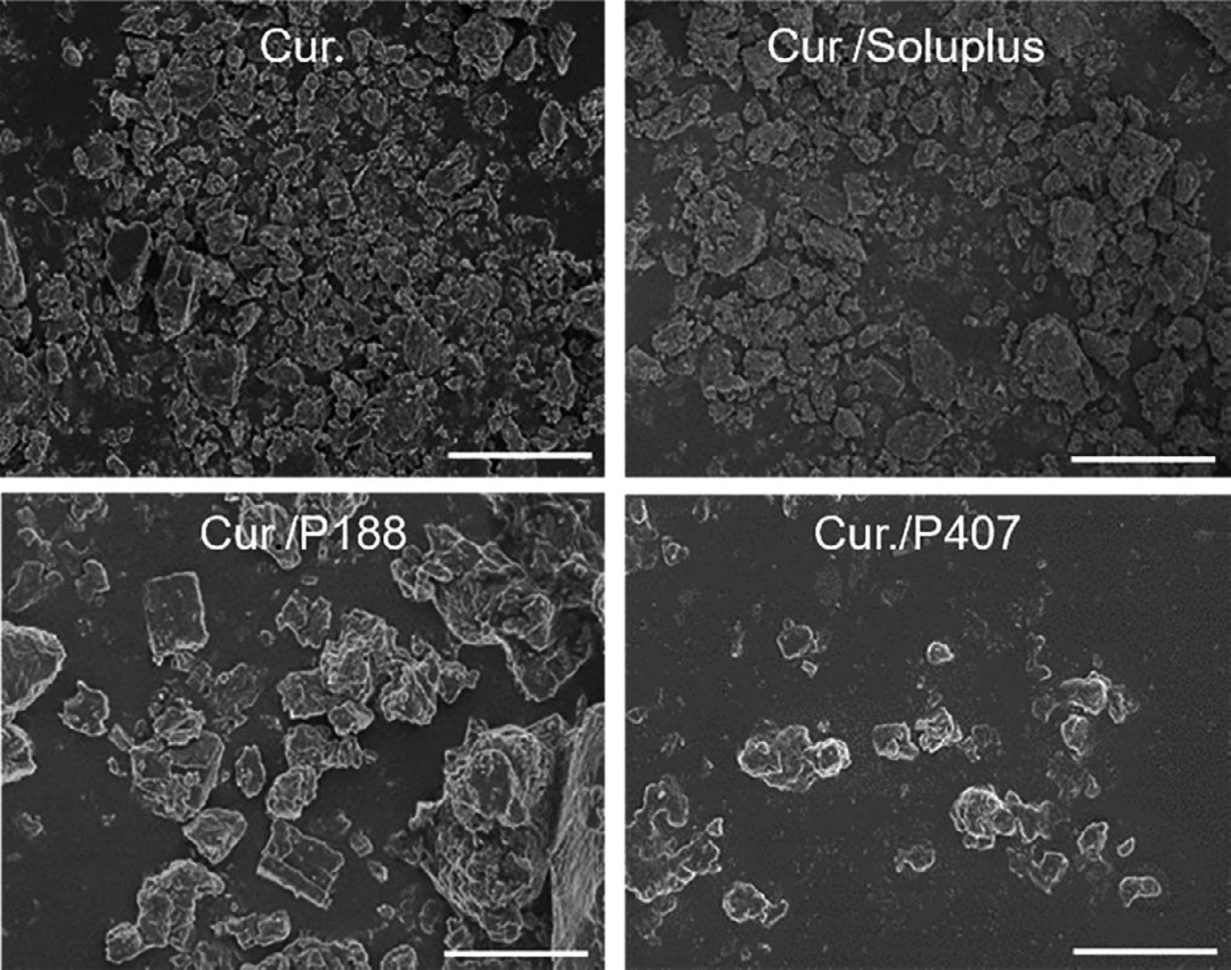
*SEM* of curcumin, Soluplus^®^ curcumin co‐grinding mixture, Kolliphor^®^ P188 curcumin co‐grinding mixture, and Kolliphor^®^ P407 curcumin co‐grinding mixture, respectively. Images are at 3,000×, bar indicates 10 μm

*SEM* showed that after grinding curcumin with P407, Soluplus, and P188, the surface morphology did not change significantly, indicating that no solid dispersions and other preparation forms were formed, which was beneficial to the long‐term stability of the mixture of curcumin and excipients. As displayed in Figure 2, surface morphology is irregular particle aggregation.

### 3.5 Dissolution test in vitro

**FIGURE 3:**
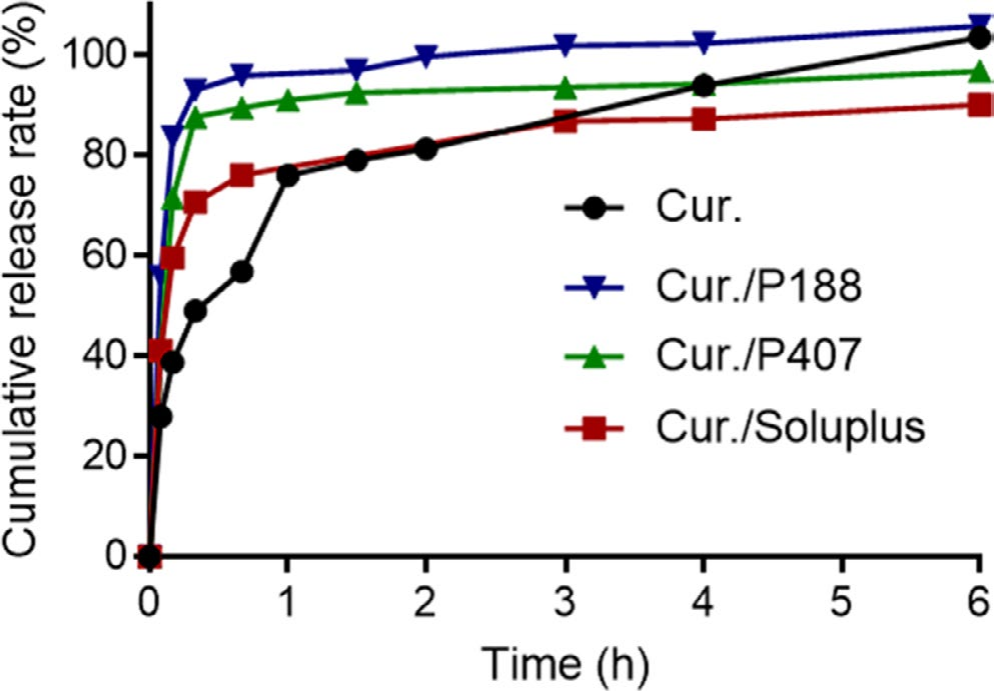
The release of curcumin from Soluplus^®^ curcumin co‐grinding mixture, Kolliphor^®^ P188 curcumin co‐grinding mixture, Kolliphor^®^ P407 curcumin co‐grinding mixture, and curcumin in 900 ml 0.5% sodium dodecyl sulfate (SDS) solution at 75 rpm and 37°C during 6 hr

The results of the dissolution experiment of curcumin API, cur./Soluplus, cur./P407, and cur./P188 after grinding were shown in Figure 3. At the first 5 sampling points (5 min, 10 min, 20 min, 40 min, and 1h), the dissolution of curcumin in cur./Soluplus co‐grinding mixture, cur./P407 co‐grinding mixture, and cur./P188 co‐grinding mixture was significantly accelerated due to the presence of excipients. In group cur./P188 co‐grinding mixture, the dissolution of curcumin reached over 80% at 10 min and over 90% at 20 min. Based on the above results, we chose P407 and P188 groups for subsequent experiments (Figure 3 and Table 2).

**TABLE 2:** Cumulative release rate of curcumin (Cur.) from different co‐grinding mixture in the first 40 min (%)

Time/min	Cur.	Cur./P188	Cur./P407	Cur./Soluplus
5	28.002	55.851	41.231	41.157
10	38.775	83.755	71.566	59.584
20	49.129	93.113	87.659	70.687
40	56.874	95.893	89.540	76.089

### 3.6 Cell tight junction test (ZO‐1 cell immunofluorescence test)

**FIGURE 4:**
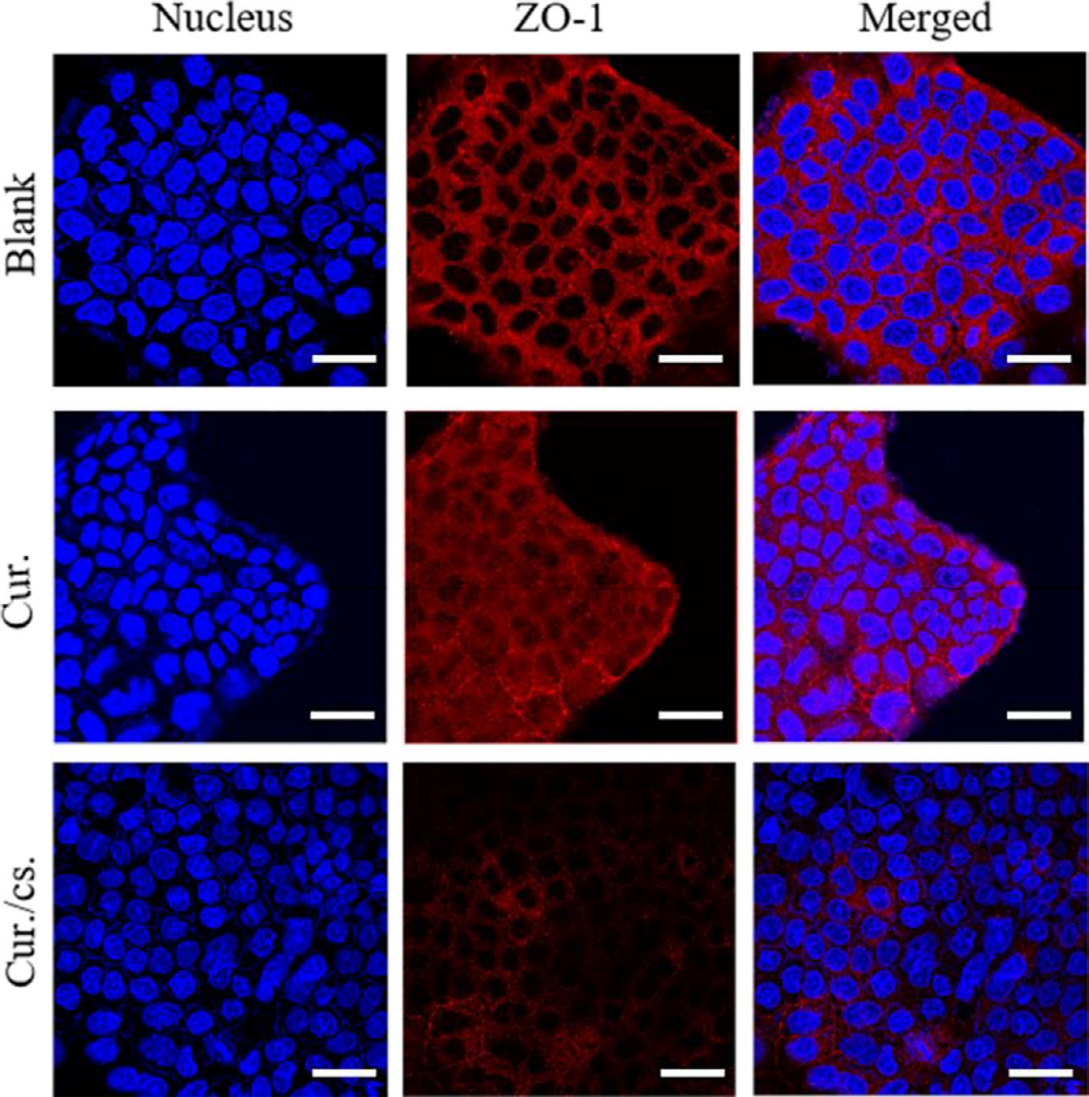
The effect of chitosan on cell tight junctions. Caco‐2 cells were co‐incubated with 20 μg/ml curcumin and chitosan at 37°C for 4 hr and dyed before being observed under confocal microscope. The nucleus was stained blue by DAPI, and cell tight junctions were stained red by ZO‐1 primary antibody followed by goat anti‐rabbit secondary antibody. Bar indicates 100 μm

It has been reported that chitosan can open tight connections between small intestinal epithelial cells and promote drug absorption (Thanou et al., 2001; Yeh et al., 2011). Our experimental results also confirmed the role of chitosan in opening tight junctions in Caco‐2 cells, which was beneficial for the absorption of curcumin (Figure 4). There are two ways for the material to pass through the small intestinal epithelial cells, namely the transepithelial cell pathway and the paracellular pathway (close connection). The paracellular pathway is a complex group of structures controlled by the tight connection of epithelial cells and the physical barrier around the cells formed by adjacent cell membrane. Compared with the BLANK group, the cells in the curcumin group were tightly connected and showed a network structure. It can be obviously seen that the network structure of cells in the chitosan and curcumin co‐grinding group was broken and the tight connections between the cells were opened, indicating that transmembrane transport is achieved by opening the tight junction structure between cells.

### 3.7 Cell uptake and cell entry mechanism study

**FIGURE 5:**
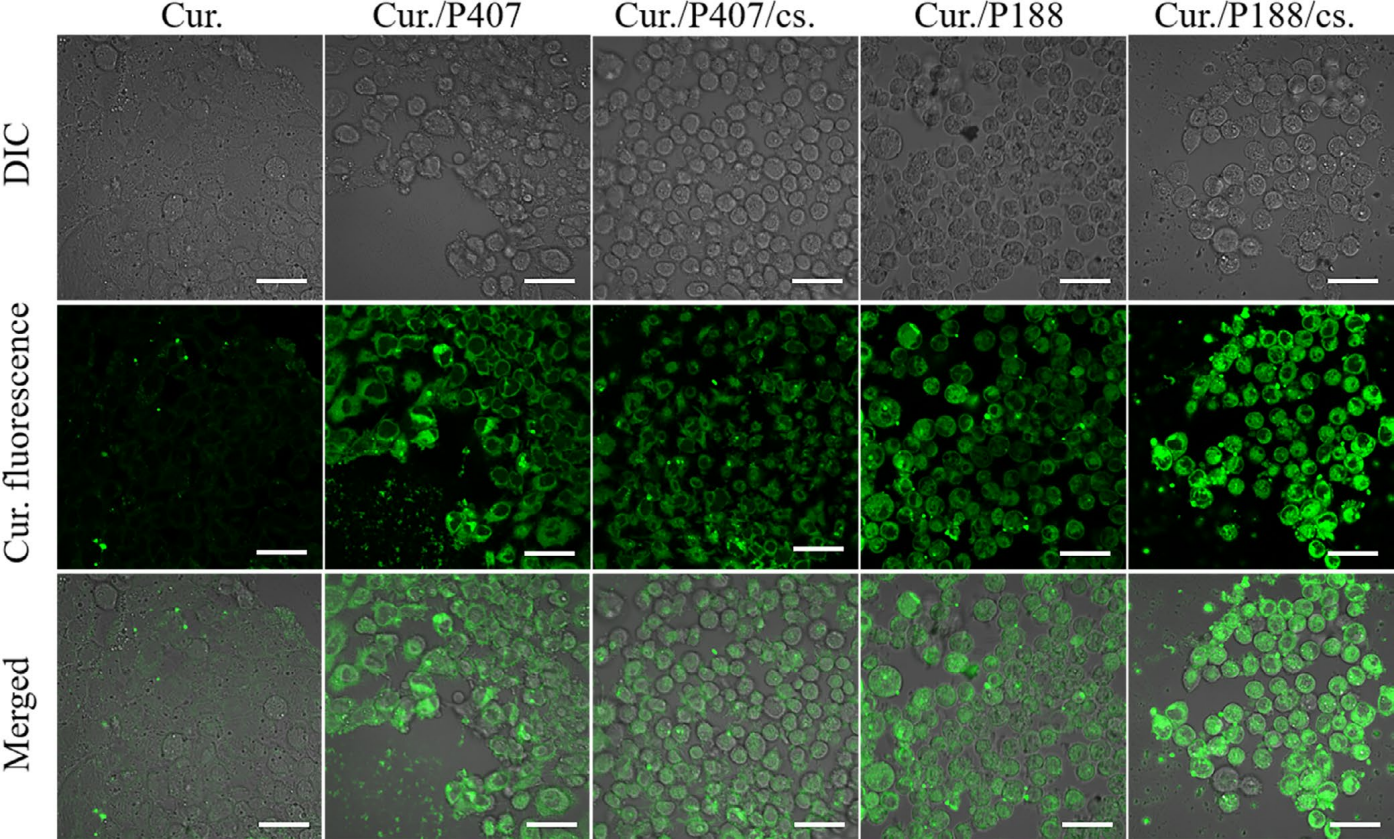
Uptake of curcumin in Caco‐2 cells. The cells were exposed to Cur., Cur./P407, Cur./P407/cs., Cur./P188, and Cur./P188/cs. co‐grinding mixture at 37°C for 4 hr, respectively, and then being visualized by confocal microscopy. Images are at 60×; bar indicates 100 μm

**FIGURE 6:**
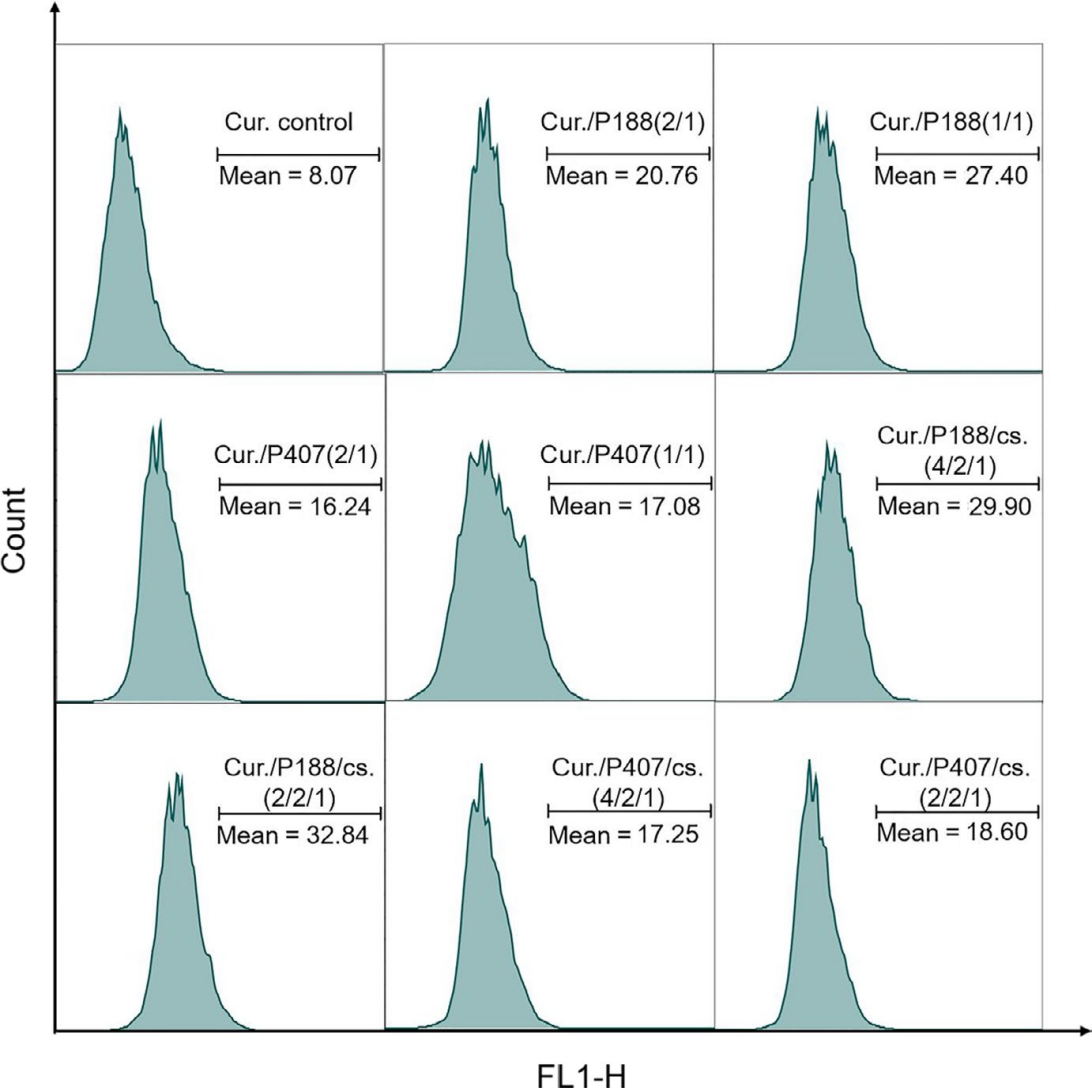
Cellular uptake of curcumin in three‐cell model: Caco‐2 cells and HT‐29 cells were seeded in 6‐well plates at densities of 7 × 10^4^ cells/ml and 3 × 10^4^ cells/ml, respectively, cultured for 2 weeks. In the last 3 days, Raji‐B cells were inoculated at the density of 1 × 10^4^ cells/ml. The cells were exposed to Cur., Cur./P188(2:1), Cur./P188(1:1), Cur./P407(2:1), Cur./P407(1:1), Cur./P188/cs.(4:2:1), Cur./P188/cs.(2:2:1), Cur./P407/cs.(4:2:1), and Cur./P407/cs.(2:2:1) co‐grinding mixture at 37°C for 4 hr, respectively (curcumin concentration: 20 μg/ml). The cells were then washed with ice‐cold PBS for 3 times and trypsinized, finally examined by flow cytometric analyses (BD FACSCalibur, USA). Mean value means curcumin fluorescence

Curcumin itself can emit green fluorescence under excitation. Under the same fluorescence parameters, we observe the intensity of green fluorescence to reflect the uptake of curcumin by Caco‐2 cells. It can be seen in Figure 5 that compared with the curcumin control group, the green fluorescence in the other four groups (Cur./P407, Cur./P407/cs., Cur./P188, and Cur./P188/cs. co‐grinding mixture) was significantly enhanced, indicating that the cells had the more uptake of curcumin. The results proved that the excipients can help curcumin enter Caco‐2 cells (Figure 6).

Further study on the intake of curcumin calculated by flow cytometry proved the following findings: (1) When the ratio of curcumin and excipients (P407 or P188) (m:m) is 4:2 or 2:2, P188 is better than P407 in increasing curcumin uptake. (2) The ratio of curcumin and excipients (P407 or P188) (m:m) 2:2 is not much better than 4:2 in promoting the uptake of curcumin cells, when considering the drug load, we tend to choose 4:2. (3) Cellular uptake of curcumin is not improved very much after adding chitosan whether the ratio of curcumin and excipients (P407 or P188) (m:m) is 4:2 or 2:2. Moreover, since chitosan is only soluble in acid water, it cannot be well dispersed in the solvent when it is administered with curcumin and P407/P188 in the gavage in consideration of the heterogeneity and instability of the preparation, it is abandoned for further exploration. And the data from our other cell uptake experiments also confirm that curcumin uptake increased by 22.89, 3.79, and 11.11 times, respectively, after the addition of p‐gp‐specific inhibitor verapamil (300 μg/ml), energy‐dependent transport inhibitor sodium azide (1 mg/ml), and cyp3a4‐specific inhibitor ketoconazole (10 μg/ml), indicating that the uptake of curcumin was influenced by p‐gp and cyp3a4 enzyme.

From the above experimental results, curcumin: P407 = 2:1 (m:m) co‐grinding mixture and curcumin: P188 = 2:1 (m:m) co‐grinding mixture were finally selected for pharmacokinetic experiments. And in order to explore the degree of increase in curcumin solubilization (the solubilization limit is completely soluted) in its absorption in vivo, we also added an experimental group of which curcumin was completely dissolved.

### 3.8 Pharmacokinetics study of curcumin

**FIGURE 7:**
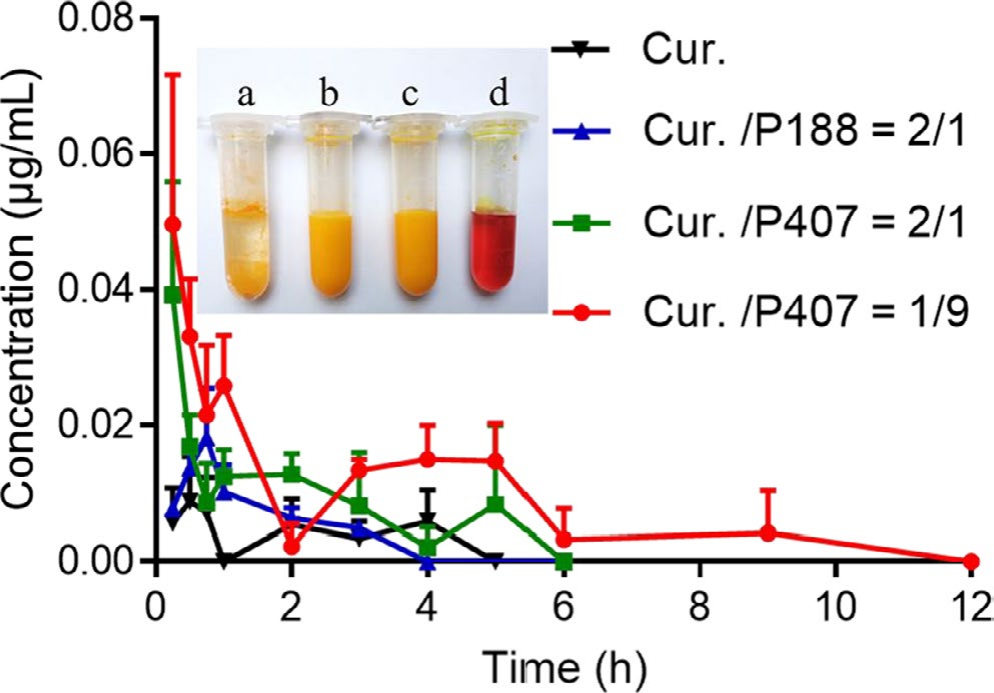
The concentration of curcumin in plasma collected from the infraorbital venous plexus of rats (*n* = 3) orally administered the curcumin formulations (a. Cur, b. Cur./P188 = 2/1 co‐grinding mixture, c. Cur./P407 = 2/1 co‐grinding mixture, and d. Cur./P407 = 1/9 dissolved in physiological saline). Values were expressed as mean ± *SD*

The curcumin level in plasma isolated from the infraorbital venous plexus of rats was recorded in Figure 7, and their pharmacokinetic parameters were calculated in Table 3 by Kinetica4.4—non‐compartmental: a, Curcumin was almost insoluble in physiological saline. b, c, Curcumin evenly dispersed in physiological saline, formatting a yellow cloudy suspension. d, Curcumin was completely dissolved in physiological saline to form an orange transparent solution. F_rel_ = AUC _co‐grinding mixture_/AUC _cur._×100%. The relative bioavailability (F_rel_) of the Cur./P407 co‐grinding mixture and Cur./P188co‐grinding mixture were 309% and 163%, respectively, compared with the curcumin API. Although at the cellular level, P188 had a more significant effect on promoting the uptake of curcumin, P407 had a better effect in animal experiments, possibly because at the cellular level, P188 could inhibit the p‐gp (Gupta et al., 2015), which was more beneficial to the uptake of curcumin. However, the better solubilization effect of P407 in rats prevailed, leading to a greater increase in bioavailability. It is worth noting that even if curcumin were completely dissolved, its relative bioavailability would be only 618%.

**TABLE 3:** Pharmacokinetic parameters (AUC_0‐∞_ and relative bioavailability) of different curcumin formulations

Samples	Curcumin dose (mg/kg)	AUC_0‐∞_ (h)*(μg/ml)	Relative bioavailability（%）
Cur.	50	0.01509 ± 0.00571	/
Cur./P188 = 2/1	50	0.02463 ± 0.00198	163.22
Cur./P407 = 2/1	50	0.04669 ± 0.02015	309.41
Cur./P407 = 1/9	50	0.09326 ± 0.02897	618.03

Note

AUC_0‐∞_, area under the curve from 0 hr to ∞. AUC data are expressed as mean ± *SD*.

## 4 CONCLUSION

In this study, partial mechanisms of curcumin ingested by intestinal epithelial cells were described, and curcumin formulations were investigated (excipients and curcumin were mixed and co‐grinded to make a mixture). Kolliphor^®^ P407 with excellent performance was screened, which significantly improved the solubility and dissolution of curcumin, increased intestinal epithelial cells uptake, enhanced oral bioavailability, and increased cytotoxicity to mammary carcinoma 4T1 (Fig S2, S3). The preparation has high drug loading, good safety, and flexible form, which can also be further used in combination with metabolic inhibitors such as piperine and further processed into various dosage forms appropriate for different populations. Being simple and cost‐effective, the prescription process is suitable for industrial production.

In addition, our results showed that the relative bioavailability would be only 618% even if curcumin was completely dissolved. To further improve the oral bioavailability of curcumin, more consideration should be given to protecting curcumin from complex biochemical environments in the gastrointestinal tract such as pH, ions, and metabolic enzymes. This research may lay a good foundation for further development of subsequent curcumin oral preparations.

## 5 ETHICAL REVIEW

This study was approved by the ethics committee of Zhejiang University.

## INFORMED CONSENT

Written informed consent was obtained from all study participants.

## CONFLICT OF INTEREST

The authors declare no conflict of interest.

## Supporting information

Fig S1‐S3

## Data Availability

The data that support the findings of this study are available from the corresponding author upon reasonable request.
