# Supplementary material for: Highly bioavailable curcumin preparation with a co‐grinding and solvent‐free process
Source: Food Sci Nutr. 2020 Oct 7;8(12):6415–25. doi: 10.1002/fsn3.1930 (PMC7723189; doi:10.1002/fsn3.1930)
Supplement: Supplementary file 1 — Fig S1‐S3 [file FSN3-8-6415-s001.docx]

**Supporting Information**

Highly Bioavailable Curcumin Preparation with A Co-Grinding and Solvent-Free Process

Yiying Lu^1^, Mengting Lin^1^, Jiancheng Zong^2^, Lei Zong^2^, Zhen Zhao^3^, Shanglong Wang^2,*^, Zengliang Zhang^2,4*^, Min Han^1,*^

1 Institute of Pharmaceutics, College of Pharmaceutical Sciences, Zhejiang University, Hangzhou, 310058, People’s Republic of China

2 Chenland Nutritionals, Inc., Irvine, California 92064, USA

3 Pathology and Laboratory Medicine, Weill Cornell Medicine, New York, NY 10065, USA

4 Traditional Chinese Medicine College, Inner Mongolia Medical University, Inner Mongolia 010110，China

**Corresponding author**

Min Han: hanmin@zju.edu.cn

Zengliang Zhang: zhangzengliang1978@outlook.com

Shanglong Wang: mwang@chenland.com

**Methods**

**Advantages of grinding/ the difference between "micronized" and "non-micronized"**

We investigated the dissolution differences between directly mixing mixture(without grinding/ non-micronized) and co-grinding mixture(micronized). The dissolution medium was 900 mL of a 0.5% sodium dodecyl sulfate (SDS) solution. The rotation speed is 50 rpm and the temperature is 37ºC. 15 mg of Kolliphor^®^ P407 directly mixing mixture (without grinding) and co-grinding mixture(Cur./P407=2/1), and 10 mg curcumin API were put into different dissolution tanks, which were sampled 3 ml in the same position with a sampling needle for 5 min, 10 min, 20 min, 40 min, 1 h, 1.5 h, 2 h, 3 h, 4 h, 5 h, 6 h, and immediately supplemented dissolution cylinder with isothermal and isovolumetric media (0.5% SDS). The removed samples were filtered by 0.22 μm microporous filter membranes and analyzed by HPLC.

**Biological activity test in vitro/ toxicity in 4T1 cells**

Mammary carcinoma 4T1 cells were cultured in 96-well plates (5×10^3^ cells per well) with DMEM complete medium. After overnight incubation, the cells were treated with cur. or cur. /P407=2/1(cur. concentration was 50,100,150 and 200 μM). After 24 h incubation, the cells were rinsed 3 times with PBS and incubated with 100 μL culture solution (including 10 μL CCK-8) for another 1 h. The optical density (OD) of each sample was measured by a microplate reader (ELx 800; Bio-Tek., USA) at 450 nm.

We further carried out crystal violet staining experiment to intuitively observe the cytotoxicity of Cur. and Cur./407=2/1. The 4T1 cells were cultured in 6-well plates with DMEM complete medium. After overnight incubation, the cells were treated with cur. or Cur./P407=2/1(cur. concentration was 200 μM). After 24 h incubation, the cells were rinsed 3 times with PBS and fixed with 4% paraformaldehyde for 15 minutes, then washed with distilled water for 2 minutes twice. After that crystal violet staining solution was added to each well and stained for 15 minutes. The cells were observed and took pictures after washed with distilled water thoroughly.

**Results**


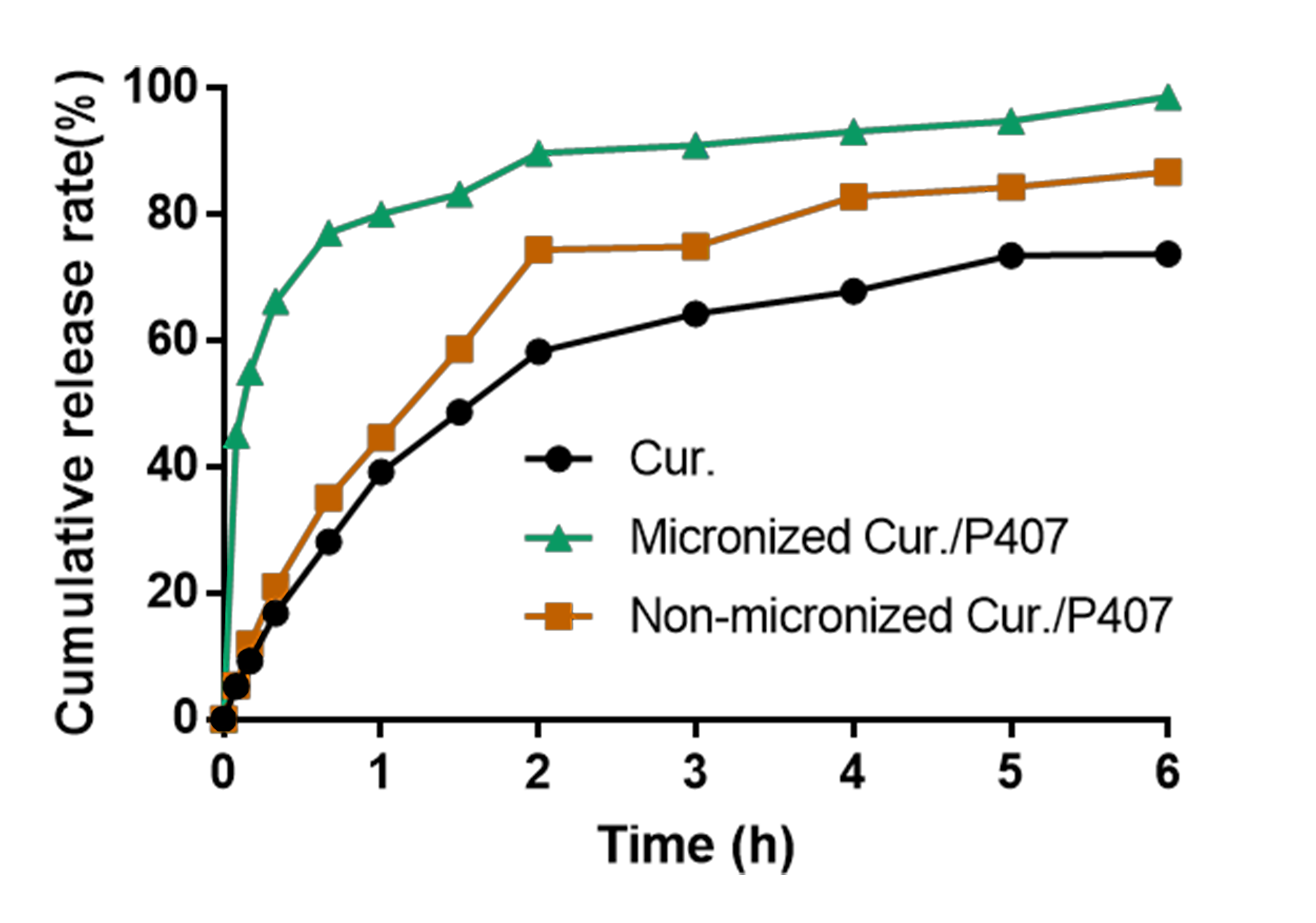


**Fig S1.** The release of curcumin from P407 curcumin mixture(without grinding/ non-micronized) ,co-grinding mixture(micronized) and curcumin in 900 mL 0.5% sodium dodecyl sulfate (SDS) solution at 50 rpm and 37ºC during 6 h.

**
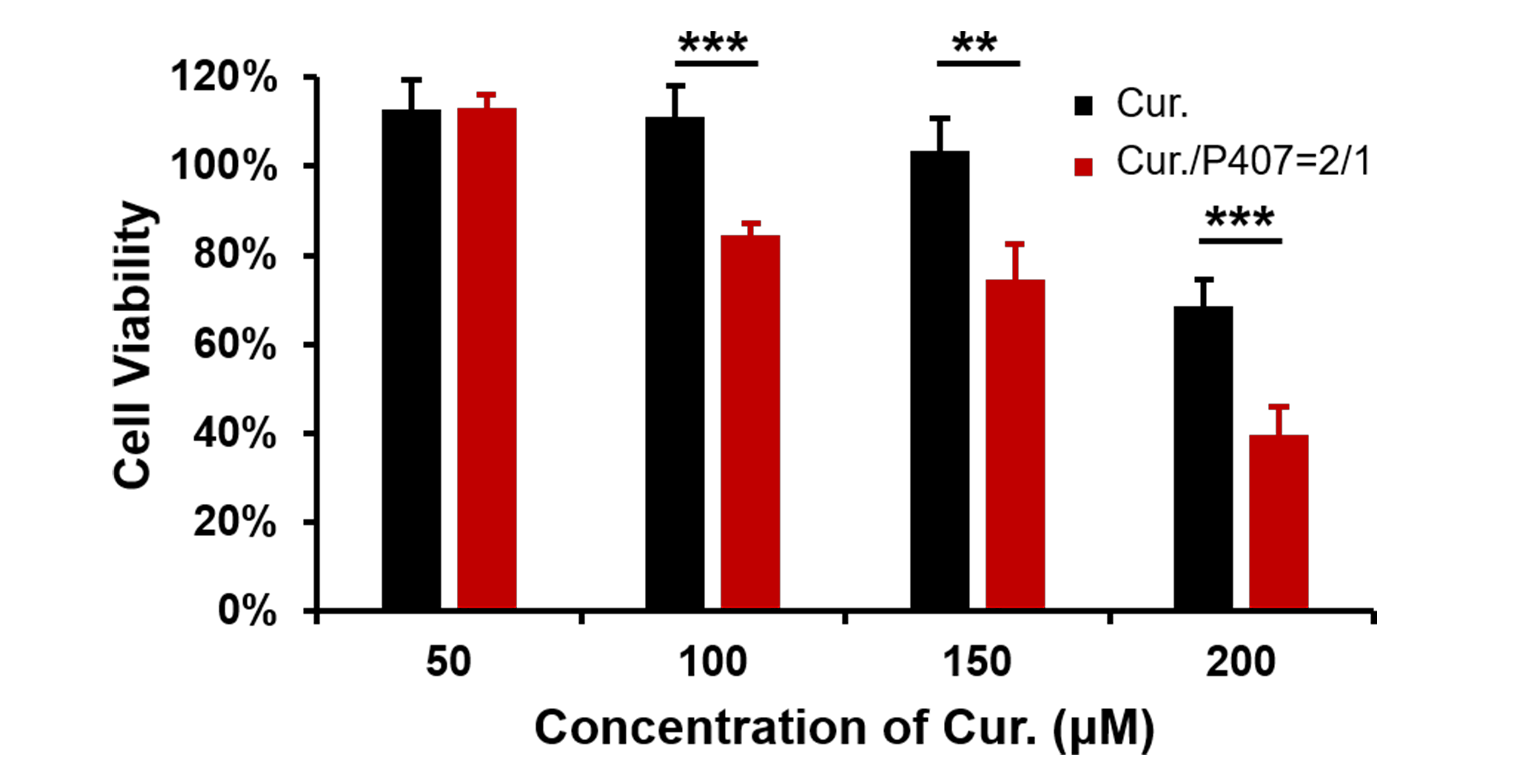
**

**Fig S2.** Cytotoxicity of curcumin and Kolliphor^®^ P407 curcumin co-grinding mixture at different concentrations to mammary carcinoma 4T1 determined by CCK-8 assay. Data are presented as means ± standard deviation, n=5.

**
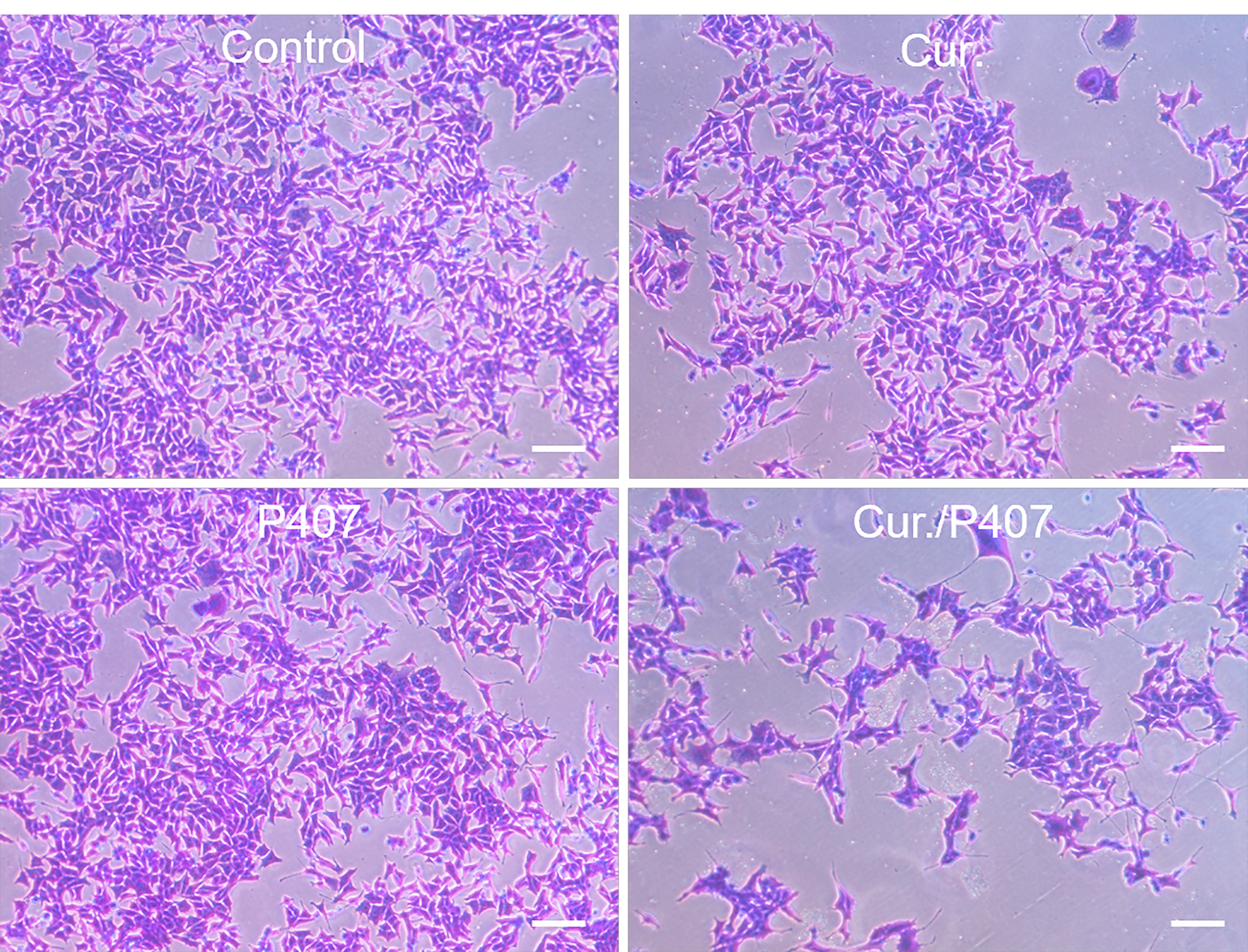
**

**Fig S3.** Cytotoxicity of curcumin and Kolliphor^®^ P407 curcumin co-grinding mixture at the same concentration(200 μM) to mammary carcinoma 4T1 showed by crystal violet staining. bar indicates 100 μm.
